# Orthogeriatric co-management improves the outcome of long-term care residents with fragility fractures

**DOI:** 10.1007/s00402-016-2543-4

**Published:** 2016-08-08

**Authors:** M. Gosch, Y. Hoffmann-Weltin, T. Roth, M. Blauth, J. A. Nicholas, C. Kammerlander

**Affiliations:** 1 Department of Geriatrics, Paracelsus Medical Private University Nuremberg, Hospital Nuremberg, 90408 Nuremburg, Germany; 2 Department of Internal Medicine I, Gastroenterology, Endocrinology and Metabolism, Medical University of Innsbruck, Innsbruck, Austria; 3 Department of Trauma Surgery and Sports Medicine, Medical University of Innsbruck, Innsbruck, Austria; 4 Rochester, Division of Geriatrics, University of Rochester Medical Center, Rochester, NY USA; 5 Department for Trauma-, Hand- and Plastic Surgery, LMU Munich, Munich, Germany

**Keywords:** Fragility fracture, Hip fracture, Long-term care patients, Outcome, Orthogeriatric co-management

## Abstract

**Background:**

Fragility fractures are a major health care problem worldwide. Both hip and non-hip fractures are associated with excess mortality in the years following the fracture. Residents of long-term nursing homes represent a special high-risk group for poor outcomes. Orthogeriatric co-management models of care have shown in multiple studies to have medical as well as economic advantages, but their impact on this high-risk group has not been well studied.

**Objective:**

We studied the outcome of long-term care residents with hip and non-hip fractures admitted to a geriatric fracture center.

**Methods:**

The study design is a single center, prospective cohort study at a level-I trauma center in Austria running a geriatric fracture center. The cohort included all fragility fracture patients aged over 70 admitted from a long-term care residence from May 2009 to November 2011. The data set consisted of 265 patients; the mean age was 86.8 ± 6.7 years, and 80 % were female. The mean follow-up after the index fracture was 789 days, with a range from 1 to 1842 days. Basic clinical and demographic data were collected at hospital admission. Functional status and mobility were assessed during follow-up at 3, 6, and 12 months. Additional outcome data regarding readmissions for new fractures were obtained from the hospital information database; mortality was crosschecked with the death registry from the governmental institute of epidemiology.

**Results:**

187 (70.6 %) patients died during the follow-up period, with 78 patients (29.4 %) dying in the first year. The mean life expectancy after the index fracture was 527 (±431) days. Differences in mortality rates between hip and non-hip fracture patients were not statistically significant. Compared to reported mortality rates in the literature, hip fracture patients in this orthogeriatric-comanaged cohort had a significantly reduced one-year mortality [OR of 0.57 (95 % CI 0.31–0.85)]. After adjustment for confounders, only older age (OR 1.091; *p* = 0.013; CI 1.019–1.169) and a lower Parker Mobility Scale (PMS) (OR 0.737; *p* = 0.022; CI 0.568–0.957) remained as independent predictors. During follow-up, 62 patients (23.4 %) sustained at least one subsequent fracture, and 10 patients (3.4 %) experienced multiple fractures; 29 patients (10.9 %) experienced an additional fracture within the first year. Nearly, half (47.1 %) regained their pre-fracture mobility based on the PMS.

**Conclusion:**

Despite the generally poor outcomes for fragility fracture patients residing in long-term care facilities, orthogeriatric co-management appears to improve the outcome of high-risk fragility fracture patients. One-year mortality was 29.4 % in this cohort, significantly lower than in comparable trials. Orthogeriatric co-management may also have positive impacts on both functional outcome and the risk of subsequent fractures.

## Introduction

Fragility fractures are a major health care problem worldwide. Due to increasing life expectancy and other associated demographic changes, the incidence of fractures and post-fracture disability appear certain to increase [1]. Fragility fractures are typically caused by osteoporosis, primarily affecting postmenopausal women, but also older men. Four out of every 10 white women age 50 or older in the United States will experience a hip, spine, or wrist fracture sometime during their lives, while 13 % of white men will suffer a similar fate [2]. Both hip fractures and non-hip fractures are associated with excess mortality in the years following the fracture [3].

The increased mortality risk lasts for 5–10 years post-fracture but is most pronounced in the first 3–6 months after sustaining a hip fracture [4, 5]. The reasons for this increased mortality risk are poorly understood [4]. Despite advances in surgical and medical care, the excess mortality of hip fracture patient remains high and has not improved over the last decade [6]. Excess mortality after hip fracture may be linked to complications following the fracture, such as pulmonary embolism, infections, and heart failure. Risk factors associated with falls and additional osteoporotic fractures may contribute to high mortality rates [7, 8]. Individual characteristics of persons sustaining a hip fracture likely play an important role, e.g., low-bone density is associated with increased non-trauma mortality, even without fractures [9]. Poor functional status is also independently linked to poor outcomes; impairments in daily activities and low-mobility scores are associated with a higher mortality [10].

Residents of long-term nursing homes represent a high-risk group for both mortality and poor functional outcomes. They are twice as likely to sustain hip fractures, and their post-fracture outcomes are worse than among community dwellers [11–13]. Many studies on hip fracture patients exclude nursing home residents or are limited by small sample size, single center design, and lack of data on functional outcomes. There is little published data looking at this cohort in particular and the impact of non-hip fragility fractures. While orthogeriatric co-management models of care have been shown in multiple studies to have medical as well as economic advantages [14, 15], there are little data to assess the impact of co-management on the long-term outcomes of this high-risk group of residents. Our study is the first to focus on the outcomes of long-term care residents after a fragility fracture (hip and non-hip) initially treated under an orthogeriatric co-management model.

## Patients and methods

### Study design

The present study is a prospective cohort study. It was done at a level-I trauma center in Austria running a Geriatric Fracture Center focused on fragility fracture patients. The Geriatric Fracture Center is characterized by an orthogeriatric co-management model [16].

No institutional review and approval was necessary in light of the clinical origin of the data, its retrospective analysis, and use of de-identified patient data.

### Study population

We included all in-hospital fragility fracture patients aged over 70 admitted from a long-term care residence from May 2009 to November 2011. The mean observation was 789 days, with a range from 1 to 1842 days. A total of 265 patients were analyzed, with a mean age of 86.8 ± 6.7 years. The majority of the cohort was female (80 %). We split the study group into two subgroups based on fracture sites (hip fractures and non-hip fractures). Non-hip fractures included humerus, wrist, rip, clavicle and sternum, vertebral, pelvis, including sacrum, lower extremities, including distal femur, and periprosthetic fractures around the knee and tibia. All patient characteristics are shown in Table 1.

**Table 1:** Baseline clinical characteristics of study population

	Overall, *n* = 256 (100 %)	Hip fracture, *n* = 130 (49.1 %)	Non-hip-fracture, *n* = 135 (50.9 %)	*p* value
Observation time (days)	789 (±561)	767 (±606)	811 (±518)	0.527
Age (years)	86.8 (±6.5)	86.5 (±6.8)	87.2 (±6.2)	0.535
Female	212 (80 %)	98 (75.4 %)	114 (84.4 %)	0.065
Surgery	175 (66 %)	122 (93.8 %)	53 (39.3 %)	<0.0001
BMI	22.9 (±4.8)	23.1 (±4.7)	22.8 (±4.9)	0.453
CCI	3.3 (±2)	3.5 (±2.1)	3.1 (±1.8)	0.228
Lachs screening	6.4 (±2.7)	6.5 (±2.9)	6.4 (±2.6)	0.914
Parker score	3.3 (±2.3)	3 (±2.3)	3.5 (±2.3)	0.066
CAM score	1.1 (1.2)	1.3 (±1.3)	0.8 (±1.1)	0.002

Values are shown as numbers with percentages in parentheses or means ± standard deviations

*BMI* body mass index, *CCI* Charlson Comorbidity Index, *CAM* confusion assessment method

### Data collection

Data collection was performed prospectively by a study nurse and four of the authors (MG, YH, TR, and CK) within the scope of a quality management project supported by the district government. The follow-up evaluations at 3, 6, and 12 months were done by a study nurse and two of the authors (TR and CK). Follow-up ended in May 2014. Hospital readmissions due to subsequent fractures were obtained from the hospital information data base, and mortality was crosschecked with the death registry from the governmental institute of epidemiology.

### Basic data

For the basic data set, we collected age, gender, fracture site, and initial treatment (surgical or non-operative) of each patient.

### Comorbidities

To evaluate and analyze comorbidities, we applied the Charlson Comorbidity Index (CCI) [17]. The CCI is valuable tool to predict the 1-year mortality for patients with up to 22 co-morbid conditions. Each condition is assigned with a score of 1, 2, 3, or 6 depending on the risk of death associated with each condition. The score is summed and given a total score which predicts mortality. The CCI was determined at admission by a geriatrician for all patients. Additional comorbidities, including acute coronary syndrome, atrial fibrillation, heart valve disease, osteoporosis, alcohol abuse, nicotine abuse, depression, pneumonia, pressure ulcers, and sarcopenia, were classified as present or not during the admission.

### Functional status

To assess the pre-fracture functional status, we used a systematized geriatric screening (SGS) described by Lachs [18]. It is a short, simple approach that can be used by physicians to routinely screen the functional status of older people. The screening is on carefully selected tests of vision, hearing, arm, and leg function, urinary incontinence, mental status, instrumental and basic activities of daily living, environmental hazards, and social support systems. It contains 15 items and can be summed-up. This tool is incorporated into the routine clinical practice of our Geriatric Fracture Center at admission.

Mobility was assessed using the Parker Mobility Score [19]. This score evaluates the patient’s ability to walk inside, outside, and when shopping or visiting family. For each question, there are four ordinal responses with a fixed count which are summed. It ranges from 0 to 9 with the maximum scores identifying independent mobility. We assessed the pre-fracture Parker Mobility Score. Functional status was assessed using the Barthel Index (BI) [21] at day 5 after admission or surgery. Delirium was assessed using the confusion assessment method on all patients during their admission [20].

Follow-up parameter:

Patients underwent a follow-up at 3, 6, and 12 months. Mortality and additional fractures were also assessed during the entire observation time. For follow-up, we assessed the functional status using the BI [21]. The BI is used to measure performance in the basic activities of daily living by scaling the presence or absence of fecal or urinary incontinence, the help needed with grooming, toilet use, feeding, transfers (e.g., from bed to chair), walking, dressing, climbing stairs, and bathing. The maximum score of 100 points indicates that the patient is independent in his basic activities of daily living, and is found to be valid outcome parameter for hip fracture patients [21].

Follow-up was conducted with the following timeline:

At 3 months: PMS, BI; at 6 months: PMS, BI;

At 12 months: PMS, BI, mortality, and additional readmissions for new fractures;

12 months to end of study: mortality, and additional fracture admissions.

### Statistical analysis

SPSS version 20.0 (2011) was used for the statistical analysis. Metric scaled data are reported as arithmetic mean ± standard deviation and categorical data as absolute frequency and percentage distribution. Non-parametric statistics (Mann–Whitney *U*-test) were used, since normality assumptions were not met for most of the outcome variables. Group effect and main condition effects were tested for significance by the Mann–Whitney *U*-test. The Chi-square test for independence was used to determine a possible relationship between two categorical variables. The significance level was defined by *p* < 0.05. Multivariate logistic regression analysis was performed to identify factors associated with one-year mortality and subsequent fractures. Bivariate analyses were based on logistic regression to generate odds’ ratios (OR) and 95 % confidence intervals (CI). The dependent variable for these analyses was one-year mortality and subsequent fractures. The independent variables were gender, age, BMI, fracture site, surgery vs. conservative treatment, pre-fracture functional status (SGS and PMS), the CCI, and CAM Score.

## Results

Our data set contained 256 long-term care residents who were hospitalized for the treatment of a fragility fracture. The majority of our patients were female (80 %). 130 (49.1 %) sustained a hip fracture, and 135 (50.9 %) sustained a non-hip fracture. All patient’s characteristics are shown in Table 1. As expected, hip fracture patients underwent significantly more frequent surgery and had a longer length of stay than non-hip fracture patients. Except for the CAM score, we found no significant differences between the two fracture groups. This cohort had a high number of comorbidities and low level of pre-fracture functionality. The most frequent comorbidities were cerebrovascular diseases (60 %), dementia (53.6 %), heart failure (52.5 %), polypharmacy (50.2 %), sarcopenia (38.5 %), urinary incontinence (29.1 %), depression (27.9 %), recurrent falls (27.5 %), malnutrition (24.2 %), hearing impairment (23.8 %), diabetes (22.7 %), atrial fibrillation (22.3 %), heart valve disease (20.8 %), chronic pain syndrome (16.6 %), and renal failure (15.5 %). The prevalence of all other diseases and geriatric syndromes was below 15 %. Mobility was severely impaired in the majority of our patients. 164 (62.6 %) had a pre-fracture PMS of lower than four points, 25 (9.4 %) were immobile with a PMS of 0, and only 15 (5.7 %) had no mobility deficit. Only one out of five were discharged to a rehabilitation unit (20.9 %). Patients admitted to a rehabilitation unit were significantly younger (85.3 ± 6.4 vs. 87.2 ± 6.4; *p* = 0.038), and had a better pre-fracture mobility (4.4 ± 2.6 vs. 3.2 ± 2.0; *p* = 0.001) and a lower CAM score (0.6 ± 0.9 vs. 1.2 ± 1.3; *p* = 0.007).

During follow-up, patients recorded their lowest mobility scores at 3 months (mean PMS 2.35 ± 1.8) followed by a small improvement over 12 months (mean 2.58 ± 1.9). The loss of mobility was the same in both hip fracture (−0.56 points) and non-hip fracture patients (–0.63 points). From 3 to 6 months, we found only a very small improvement (mean +0.12 points). However, nearly, half of the cohort (47.1 %) regained their pre-fracture mobility based on the PMS (hip fracture 43.9 %, non-hip fracture 53.6 %, and *p* = 0.402.). PMS after 12 months was not significantly associated with discharge to a rehabilitation unit.

Following admission, the lowest BI was noted on post-operative day 5 (mean 31.2 ± 21.0) with hip fracture patients demonstrating a lower BI (26.2 ± 20.3) compared to non-hip fracture patients (38.6 ± 19.9). In both fracture groups, we observed an improvement in independence from day 5 to 3 month follow-ups (overall BI improvement 17.2 points, hip fracture 19.1, and non-hip fracture 14.4). At the 6 and 12 month follow-ups, we found a negligible improvement in the BI.

187 (70.6 %) patients died during the observation time. The mean life expectancy after the index fracture was 527 (±431) days. Males had a non-significantly shorter survival time compared to female patients (458 ± 403 days vs. 546 ± 438 days; *p* = 0.322). Within the first 12 months, 78 (29.4 %) patients died, with no statistically significant differences based on fracture group. Age, low BMI, CCI, SGS, PMS, and CAM were significantly associated with 1-year mortality (Table 2). After logistic regression, including sex, age, fracture site, surgical vs. conservative treatment, LOS, BMI, CCI, SGS, PMS, and CAM, only older age (*p* = 0.013; OR 1.091; CI 1.019–1.169) and a lower PMS (*p* = 0.022; OR 0.737; CI 0.568–0.957) remained as independent predictors of one-year mortality. Figure 2 shows the one-year mortality risk in different age groups by different PMS groups.

**Table 2:** Baseline clinical characteristics of patients deceased within 12 month

	Survivors, *n* = 187 (70.6 %)	Deceased within 12 month, *n* = 78 (29.4 %)	*p* value
hip fracture	86 (46 %)	44 (56.4 %)	0.122
Age (years)	85.8 (±6.3)	89.2 (±6.3)	0.001
Female	152 (81.3 %)	60 (76.9 %)	0.419
Surgery	123 (65.8 %)	52 (66.7 %)	0.889
LOS	9.9 (±7.2)	8.4 (±6.3)	0.049
BMI	23.3 (±5.1)	22.0 (±3.9)	0.03
CCI	3.0 (±1.9)	4.0 (±2.1)	<0.0001
Lachs screening	6.2 (±2.7)	7.2 (±2.5)	0.034
Parker Score	3.5 (±2.5)	2.6 (±1.8)	0.011
CAM Score	0.9 (±1.1)	1.5 (±1.3)	0.001

Values are shown as numbers with percentages in parentheses or means ± standard deviations

*LOS* length of stay, *BMI* body mass index, *CCI* Charlson Comorbidity Index, *CAM* confusion assessment method

**Fig. 1:**
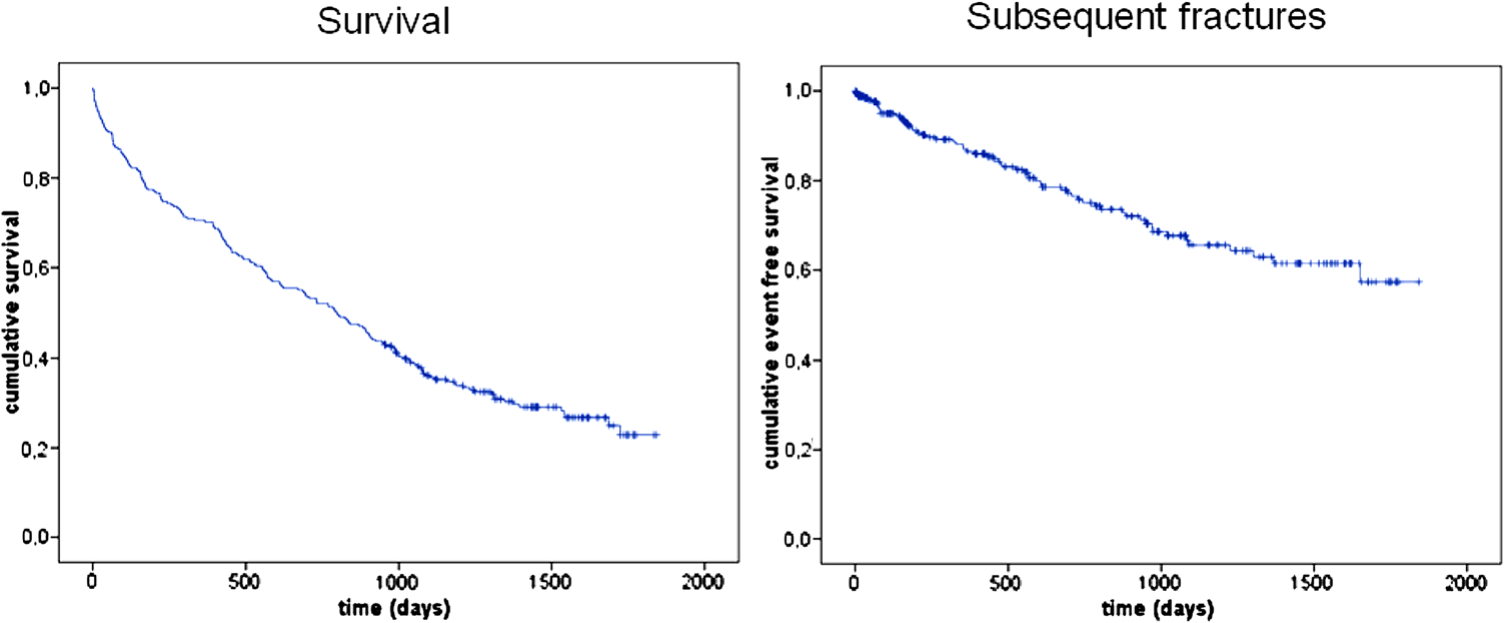
Kaplan–Meier survival and event free (subsequent fractures) curve

During follow-up, 62 (23.4 %) patients sustained one subsequent fracture and 10 (3.4 %) patients sustained more than one fracture; overall, 29 (10.9 %) patients had recurrent fractures within the first year (Fig. 1). Fracture group, surgical treatment, CCI, SGS, PMS, and CAM were significantly associated with the presence of a subsequent fracture (Table 3). After logistic regression, including sex, age, fracture group, operative vs. non-operative treatment, LOS, BMI, CCI, SGS, PMS, and CAM, none of them remained as independent predictor of the subsequent fracture.

**Table 3:** Baseline clinical characteristics of patients with subsequent fractures

	No fracture, *n* = 203 (76.6 %)	Subsequent fracture, *n* = 62 (23.4 %)	*p* value
Hip fracture	108 (53.2 %)	40 (64.5 %)	0.015
Age (years)	86.7 (±6.6)	87.1 (±6.0)	0.526
Female	162 (79.8 %)	50 (80.6 %)	0.885
Surgery	141 (69.5 %)	34 (54.8 %)	0.033
LOS	9.6 (±7.1)	9.2 (±6.8)	0.545
BMI	22.9 (±4.9)	22.9 (±4.5)	0.264
CCI	3.4 (±2.1)	2.7 (±1.6)	0.025
Lachs screening	6.7 (±2.8)	5.8 (±2.2)	0.070
Parker Score	3.0 (±2.3)	3.9 (±2.3)	0.009
CAM Score	1.2 (±1.2)	0.7 (±1.1)	0.010

Values are shown as numbers with percentages in parentheses or means ± standard deviations

*LOS* length of stay, *BMI* body mass index, *CCI* Charlson Comorbidity Index, *CAM* confusion assessment method

## Discussion

The poor outcome of the long-term care residents after a fragility fracture in usual care settings is already well known [10, 22, 23]. P. Orthogeriatric co-management models have shown beneficial effects on the outcomes of older fragility fracture patients [14, 15]. Data regarding special subgroups, such as long-term care residents, are rare. Our study is the first to focus on the long-term-care residents treated under an orthogeriatric co-management model. A mean CCI was 3.3, SGS of more than 6 and PMS of 3.3 reflect the high number of comorbidities and disabilities in our study population compared to other fragility fracture cohorts [10, 24]. 38.5 % of our patients had a CCI of 4 or higher. In a comparison cohort, Neuman et al. found a lower percentage of patients with similar CCI scores (26.6 %) [25]. As we expected, life expectancy is extremely limited in these patients. The mean survival time in our study was relatively long at 527 days as compared to the cohort studied by Neuman et al. at 377 days [25]. Compared to the results of Neuman, who described a 1-year mortality in hip fracture patients of 47 %, we found a much lower mortality rate [25]. One-year mortality in our study was 29.4 % in the overall group and 33.8 % in hip fracture patients. For hip fracture patients, we calculated a significantly reduced one-year mortality in patients treated in our orthogeriatric co-management model with an OR of 0.57 (95 % CI 0.31–0.85). Poor outcomes among long-term nursing home residents with hip fractures have previously been noted in other small trials. Berry et al. noted a mortality rate of 40 % at 1 year, Beaupre et al. noted 45 %, and Morrsion and Siu noted a 6-month mortality of 55 % [26–28]. Other than our orthogeriatric co-management model, we did not find any other reason for the lower mortality rate. However, we are not able to describe, how our model leads to a decrease of mortality. Our orthogeriatric co-management model is based on multifactorial, interdisciplinary interventions.

**Fig. 2:**
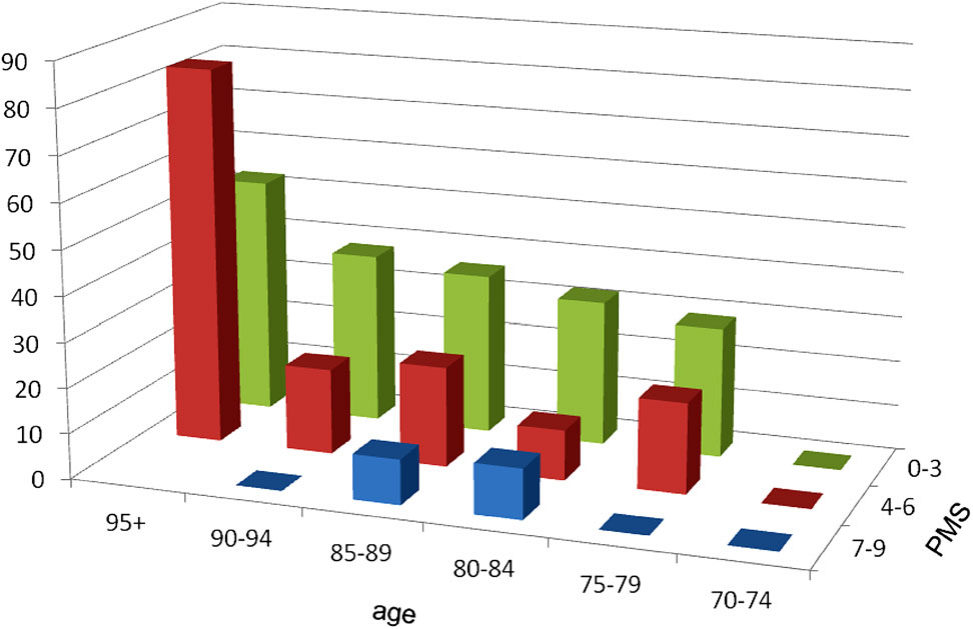
Mortality risk is shown as percentage in different age groups and level of mobility

Among our study, population's higher age and a lower PMS were the only independent risk factors for one-year mortality. Surprisingly, CCI and others factors did not remain significant predictors after adjustment for other risk factors. Older patients with a low PMS had the highest one-year mortality risk. Figure 2 shows the one-year mortality risk in different age groups and PMS groups (9–7, 6–4, 3–0 points).

Secondary fracture prevention is a main goal of orthogeriatric co-management. 23.4 % of all patients sustained a subsequent fracture during observation time, 10.9 % within the first year. Even in the group of survivors, the rate of subsequent fractures remained low at 26.9 %. The follow-up time for this cohort was long at more than 4 years (mean 1417 days, 95 % CI 954–1842 days). For comparison, we calculated 573 person years for the overall cohort. From our data, we calculated a risk of any subsequent fracture at 108 per 1000 person years. This was comparable to the result of Center et al., who assessed similar risks 90 per 1000 person years in community-dwelling men and women over the age of 80 [29]. In light of similar outcomes, despite significant differences in fracture risk, we postulate that our orthogeriatric approach likely reduces the risk of subsequent fractures. The quantity of this benefit cannot be determined without more comparable study populations or a more rigorous study design. In this study, fracture risk approximates a J-curve with the lowest rate of the subsequent fractures (20.7 %) in patients with a PMS lower than 4 and the highest in patients with a score from 7 to 9 (37 %). In long-term care patients, immobility may reduce the risk of subsequent fractures, and we found higher rates of fracture in patients with higher PMS. This could have an impact on decisions regarding osteoporosis treatment. In our study, the risk of a subsequent fracture was much lower than the mortality risk and older long-term care patients with low PMS may not benefit from osteoporosis treatment.

### Limitations

This study has several limitations. It is an uncontrolled single center setting. Our study population is a selected group of hip and non-hip fracture patients admitted to a geriatric fracture center, and may not be easily generalizable to other health care settings or communities. Furthermore, we were not able to receive information about the causes of death. However, the long-term care patients usually do not undergo autopsy. Even if we got this data, the information about the causes of death—with respect to their multimorbidity—remains uncertain and more or less scientifically doubtful. While the total number of patients is sufficient, the proportion of male patients and some subgroups is under-represented. Male patients are relatively under-represented in this cohort; this likely impaired the ability to identify any impact of gender on outcomes. Limitations also include the lack of controls and comparable cohorts for the estimation of treatment effect.

## Conclusion

Our trial is the first to evaluate both hip and non-hip fracture patients admitted from the long-term care settings to geriatric fracture center. Despite the overall high mortality and poor post-operative mobility, orthogeriatric co-management seems to be able to improve the outcome of high-risk fragility fracture patients. One-year mortality was 29.4 % and significant lower than that found in comparable trials. Orthogeriatric co-management also seems to have an impact on functional outcome and the risk of subsequent fractures. Further trials and analysis are necessary to quantify the benefit of co-management for this special group of patients, particularly in regard to how it influences the outcome.
